# Potential Role of the Fragile Histidine Triad in Cancer Evo-Dev

**DOI:** 10.3390/cancers15041144

**Published:** 2023-02-10

**Authors:** Zheyun Niu, Dongming Jiang, Jiaying Shen, Wenbin Liu, Xiaojie Tan, Guangwen Cao

**Affiliations:** 1 Shanghai East Hospital, Key Laboratory of Arrhythmias, Ministry of Education, Tongji University School of Medicine Tongji University, Shanghai 200120, China; 2 Shanghai Key Laboratory of Medical Bioprotection, Shanghai 200433, China; 3 Key Laboratory of Biological Defense, Ministry of Education, Shanghai 200433, China; 4 Department of Epidemiology, Second Military Medical University, Shanghai 200433, China

**Keywords:** fragile histidine triad, cancer evolution, genomic instability, retro-differentiation, APOBEC3

## Abstract

**Simple Summary:**

Tumor development follows an evolutionary pattern of “mutation-selection-adaptation”, characterized by exogenous oncogenic induction and endogenous replicative stress. The fragile histidine triad (FHIT) is a tumor suppressor. Aberrant transcription or reduction in the transcription and translation of the FHIT is an early event occurring in at least 50% of preneoplastic lesions and human cancers. Here, we summarized the evidence of the FHIT in cancers and evaluated the role of the FHIT in bridging macroevolution and microevolution and its functions in critical aspects of cancer evolutionary development (Cancer Evo-Dev), a theory developed to elucidate the mechanisms of non-resolving inflammation-induced carcinogenesis and develop suitable prophylactic and therapeutic options for malignant diseases.

**Abstract:**

Cancer development follows an evolutionary pattern of “mutation-selection-adaptation” detailed by Cancer Evolution and Development (Cancer Evo-Dev), a theory that represents a process of accumulating somatic mutations due to the imbalance between the mutation-promoting force and the mutation-repairing force and retro-differentiation of the mutant cells to cancer initiation cells in a chronic inflammatory microenvironment. The fragile histidine triad (FHIT) gene is a tumor suppressor gene whose expression is often reduced or inactivated in precancerous lesions during chronic inflammation or virus-induced replicative stress. Here, we summarize evidence regarding the mechanisms by which the FHIT is inactivated in cancer, including the loss of heterozygosity and the promoter methylation, and characterizes the role of the FHIT in bridging macroevolution and microevolution and in facilitating retro-differentiation during cancer evolution and development. It is suggested that decreased FHIT expression is involved in several critical steps of Cancer Evo-Dev. Future research needs to focus on the role and mechanisms of the FHIT in promoting the transformation of pre-cancerous lesions into cancer.

## 1. Introduction

Over the past few decades, cancer has become the second leading cause of human death [1], leading to enormous economic and medical burden. Cancer development represents an evolutionary process. Many attempts have been made to identify possible causes and evolutionary factors for cancer [2]. Cancers of most histological types result from persistent stimulation of both exogenous and endogenous carcinogenic factors. The endogenous contributors of carcinogenesis mainly includes ageing, genetic predisposition, metabolic syndrome, and non-resolving inflammation [3], while the exogenous contributors includes tobacco smoking, air pollution, radiation, toxins such as aflatoxin, and chronic infection with viruses and bacteria. Usually, exogenous oncogenic factors induce carcinogenesis via activating endogenous factors such as apolipoprotein B mRNA-editing enzyme catalytic polypeptide-like (APOBEC) family members and/or inactivating another group of endogenous factors including the fragile histidine triad (FHIT). Interaction of exogenous contributors and endogenous contributors should be important in understanding the mechanisms of carcinogenesis. For instance, hepatitis B virus (HBV)-induced hepatocarcinogenesis is significantly associated with genetic predisposition-determined immune selection of some HBV mutations, HBV integration into human genome, and non-resolving inflammation caused by HBV replication [4,5]. A cancer evolutionary theory presented by Henry Heng states that due to either exogenous oncogenic factors such as viral/bacterial infections, exposures to carcinogens and other stressors, and/or endogenous factors such as tissue/organs constrains or chromosomal instability, genomic chaos appears in a cell, leading to genomic disorganization, aneuploidy, and polyploid giant cancer cells. This is called the macroscopic stage of cancer evolution, or macroevolution, a discontinuous and rapid process causing the entire cell karyotype to be reorganized. This process is followed by a slower phase of continuous microevolution, or stepwise Darwinian clonal evolution, and a process of adaptation and selection then passes on new karyotypes in a gradual, slow and continuous manner, eventually evolving into cancer [6,7]. During this microevolutionary stage, the positive selection of cancer gene mutations and epigenetic factors can contribute to mutated cell growth [8]. Continuous cycling between macroevolution and microevolution via the polyploid and diploid genomes creates a highly dynamic evolving system for malignant transformation in response to endogenous and exogenous stresses [9]. The process of tumorigenesis follows an evolutionary pattern of “mutation-selection-adaptation” in affected cells [5,10]. Typically, mutations in acute inflammation are quickly cleared or repaired and do not drive cancer development [11]. In persistent external stimuli or chronic unresolved inflammation, proinflammatory molecules such as interleukin-6 (IL-6) may *trans*-activate the expression levels and/or alter the expression patterns of corresponding mutation-promoting genes including activation-induced deaminase (AID)/APOBEC3 family members and epigenetic modifying genes such as histone deacetylases (HDAC) within normal cells, resulting in multiple somatic mutations and epigenetic changes in cell cycle- and metabolism-related genes that can accumulate in the affected cells [5]. Endogenous APOBEC3 cytosine deaminases generate prevalent mutational signatures in human cancer cells [12,13]. During HBV-induced hepatocarcinogenesis, a large number of HCC-risk HBV mutations, aneuploidy, and somatic mutations emerge, which facilitates the evolutionary development of hepatocellular carcinoma (HCC). The affected cells might transform surrounding fibroblasts into cancer-associated fibroblasts (CAFs) via secreting some proinflammatory cytokines. CAFs recruit some immune inhibiting cells including M2 macrophage, myeloid derived suppressor cell, neutrophil, regulatory T (Treg) cells, and endothelial to establish the tumor microenvironment (TME) [5,10]. During HBV-induced hepatocarcinogenesis, the HBV mutations in the HBx gene and the large S gene, which are generated by incompetent immunity during the chronic infection, in turn promote the generation of pro-inflammatory TME via secreting plasminogen activator inhibitor-1 and activating STAT3 signaling pathways, respectively [14,15]. Under selection pressures of TME and accompanying hypoxia, a small number of viral or somatic mutations conferring “stemness” and survival advantage to the mutated cells are selected out. High-grade tumor development is remarkably similar to pre-embryogenic development [9]. Retro-differentiation or reverse development, which is in contrast to embryogenic development, is common in cancer development. When positive selection outweighs negative selection, some driver mutations accumulate, leading to cancer development, recurrence, and metastasis [16,17]. Finally, these cells gain stemness and strong clonal capacity through reverse differentiation and/or epithelial–mesenchymal transition (EMT), adapting to the TME [5,10,18,19]. Based on evidence obtained in HBV-induced hepatocarcinogenesis and other research, we proposed a novel theory of cancer development, namely Cancer Evo-Dev [5,10].

Throughout the process, the APOBEC3 signature mutation is a key to the signature mutation of most cancer types [12,13,20]. Aberrant expression of APOBEC3B can generate several C>U or C>T mutations in the genome that drive cancer development [21,22]. APOBEC3B mutations prefer to induce mutation in single-stranded DNA (ssDNA) [23,24]. Therefore, genes that promote the rise of ssDNA in the genome are critical in cancer evolution. Decreased FHIT expression, especially FHIT deletion, increases double-bond breaks in the genome and increases levels of ssDNA [25]. As ssDNA is an optimal substrate for the AID/APOBEC3 cytidine deaminases, the FHIT facilitates the mutagenic effect of AID/APOBEC3s. Thus, the FHIT might bridge macroevolution and microevolution, the two sequential forms of cancer evolution. As discussed late in this article, FHIT loss facilitates the development of the EMT. Thus, the role of the FHIT is indispensable in Cancer Evo-Dev.

The *FHIT* gene, located on chromosome 3p14.2, was identified in 1996 through exon capture [26]. The FHIT is a histidine triplet protein superfamily member, a diadenosine 59,5–P1, P3-triphosphate (Ap3A) hydrolase. The *FHIT* gene consists of ten exons, of which exons one to four and ten are involved only in transcription. Exons five to nine are protein codons that form an open reading frame (ORF), encoding a small protein of 147 amino acids [25]. The 5′ end contains a noncoding region, and the 3′ untranslated region has a poly-A consensus sequence and a poly-A tail. The FHIT functions as a tumor suppressor. The tumorigenicity of the FHIT-transfected cells is significantly reduced in vivo [25,27].

**Figure 1:**
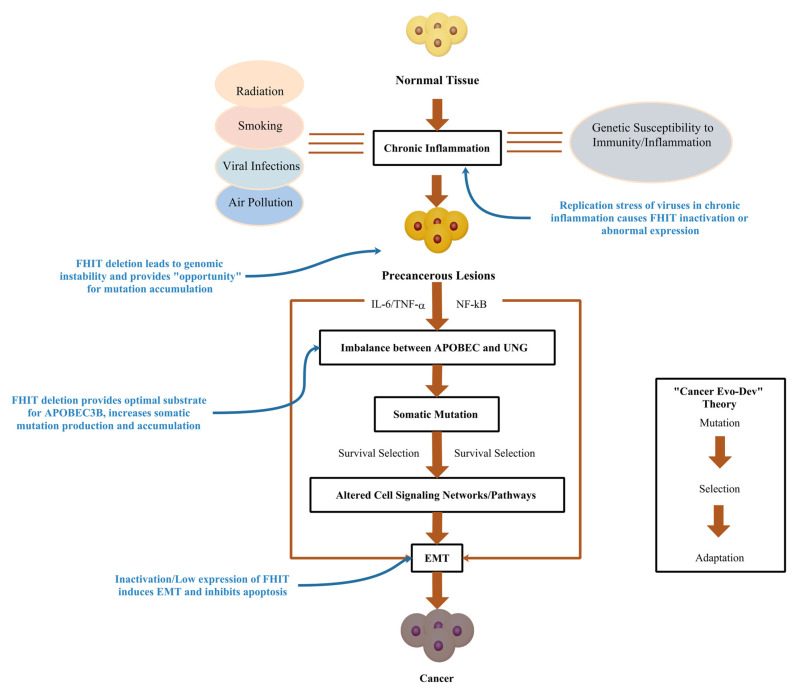
Potential Role of FHIT in Cancer Evo-Dev.

In this review, we summarized evidence of the FHIT from the perspective of cancer occurrence and the roles of the FHIT in various aspects of Cancer Evo-Dev. An early persistent chronic inflammatory environment leads to abnormal expression of the FHIT through several pathways. Aberrant expression of the FHIT causes genomic instability, including increased replication stress, and chromosomal instability, thus providing opportunities for somatic mutations. Most importantly, low expression of the FHIT provides the most suitable substrate for APOBEC3 mutations, contributing to the accumulation of many APOBEC3-related mutations in the genome. The FHIT is also involved in promoting EMT and inhibiting apoptosis, which immortalizes the mutated cells and drives the accumulation of somatic mutations. Finally, these mutations accumulate to sufficient numbers that drive the heterogeneity of tumor cells in vivo and promote cancer evolution (Figure 1).

## 2. Inactivation/Low Expression of the FHIT Gene

Tumor suppressor genes with regular expression inhibit cell proliferation and tumorigenesis. Decreased FHIT expression leads to the malignant transformation of affected cells and promotes the evolutionary development of cancer. Aberrant transcripts or decreases in the transcription and translation of the FHIT are present in at least 50% of preneoplastic lesions and human cancers, especially in esophageal, lung, liver, stomach, pancreatic, kidney, skin, breast, and cervical cancers [28,29,30]. Abnormal expression of the FHIT is also evident in hyperplastic lesions [31,32], suggesting that the inactivation of the FHIT plays a vital role in inhibiting the formation of early preneoplastic and premalignant lesions. There are three basic pathways leading to the aberrant expression of the FHIT: replication stress, loss of heterozygous (LOH), and CpG methylation at the promoter region.

### 2.1. The FHIT/FRA3B Is Sensitive to Replication Pressure

The FHIT gene spans a 2-Mb genome and contains one of the most common fragile sites (CFSs), FRA3B [33]. FRA3B deletions are frequently observed in cancer cells due to endogenous or exogenous damage. The corresponding deletion of the FHIT protein after FRA3B deletion may predict malignant tumor formation [34,35]. Studies show that the changes in the FHIT expression are associated with FRA3B deletion [36]. CFSs are preferentially unstable in pre-cancerous lesions or preneoplastic stages, leading to altered gene function during tumorigenesis or progression [37]. CFSs are genomic loci prone to the formation of breaks or gaps on metaphase chromosomes, which are characterized by AT-rich sequences, complex replication, and transcriptional repression due to late replication timing and large transcription units [38]. The completion of replication at CFS occurs very late in the cell cycle at mitosis through a process termed mitotic DNA synthesis (MiDAS) [39]. The transcriptional process of oversized genes often extends well beyond one cell cycle and even into the next, leading to simultaneous replication and transcription and creating “fragility” [40]. Typically, CFSs are stabilized by relaxing the DNA superhelix and restricting the formation of R-loops to resolve transcription–replication collision [37,41,42]. CFSs are hypersensitive to replication stress. When stimulated by exogenous or endogenous factors, they are highly susceptible to breakage [43]. Early pre-cancerous lesions create a continuous environment of replication stress. FRA3B is one of the most common CFSs, and exposure to constant stress predisposes it to more breakage due to replication–transcription conflicts.

### 2.2. Repeated Breakages and Repairs Cause Loss of Heterozygosity in the FHIT

After DNA damage, DNA repair is performed by the flanking, long interspersed nuclear element 1. Aphidicolin blocks replication forks in replicating S-phase cells, resulting in DNA double-strand breaks (DSBs). The extent of DNA end excision is the primary factor determining whether repair proceeds via non-homologous end joining (NHEJ) or homologous recombination. NHEJ occurs at any time throughout the cell cycle, unlike homologous recombination, which occurs mainly in the S/G2 phase [38,44]. Prolonged and repeated breaks and repairs cause improper segregation of chromosomes during mitosis, resulting in LOH [30,45]. LOH is evident at the FHIT in more than 90% of lung tumors, and at least one allele of three markers (D3S1300, D3S1312, or D3S1313) is lost in the primary tumor and the corresponding bronchoalveolar lavage fluid [46]. There are 11 recurring breakpoint/repair regions, and deletions occur mainly between intron three (near exon four) and intron five (150 kb distal to exon five) [47]. In addition, splicing events also affect the FHIT expression, usually appearing as exon 4–6 deletions caused by aberrant splicing in exons 3–7 and exon 4–8 deletions caused by aberrant splicing in exons 3–9. 82% of colorectal adenocarcinomas have the FHIT deletions, fragment insertions, point mutations, or alternative splicing in exon ten [48]. Notably, partial deletion of the FRA3B sequence does not reduce the occurrence of breakages and instability within the remaining sequences at the FHIT gene, with the highest frequency of chromosomal breaks and gaps occurring in the 300-kb interval between exon four and D3S1300 [49]. Depletion of FHIT neither activate the DNA damage response nor cause cell cycle arrest, allowing continued cell proliferation and ongoing chromosomal instability. LOH can be fatal in inactivating the function of the FHIT, which prevents the FHIT from acting as a “gene caretaker”, leading to a significant increase in genomic instability.

### 2.3. CpG Methylation at the FHIT Promoter Region

Methylation at the FHIT gene promoter is also an essential mechanism of the promoter inactivation. The methylation tends to suppress the expression of the FHIT, allowing the generation and accumulation of mutations in tumors that drive the evolutionary development of cancer [34]. In locally advanced lung cancer, FHIT mRNA expression is frequently absent via the CpG methylation [50]. For Asian populations, aberrant methylation of the FHIT can be used as a potential diagnostic biomarker for non-small cell lung cancer [50]. The FHIT^low^/pHER2^high^ signature is associated with the higher size of tumors, lymph node involvement, and late TNM stages, serving as an independent predictor of poor prognosis in lung adenocarcinoma whilst also being predictive of a poor response to immune checkpoint inhibitors in advanced lung cancer [51]. Double allelic inactivation of the FHIT gene and its complete silencing of the FHIT gene by heterozygous deletion has also been found in breast cancer [52]. Exposure to ambient air pollution throughout life may be associated with DNA methylation of some tumor suppressor genes such as FHIT in breast tumor tissue [53]. In esophageal cancer, variations in the FHIT gene mainly arise from a loss of exon five or eight or hypermethylation of the FHIT promoter [54]. Nevertheless, it remains unknown how the CpG methylation is induced. Because inactivation is often observed at the early stages of pre-cancerous lesions, it is likely to be caused by chronic inflammation or activated oncogenes.

## 3. Aberrant Expression of the FHIT Contributes to the Genome Chaos

Chromosomal polyploidy or aneuploidy resulting from genomic instability is often observed in pre-cancerous lesions, producing viable altered cells with new genomes [55,56]. These chromosomal re-patterning may have exactly the same effect as the accumulation of mutations. Most macroevolution is eliminated by systemic constraints. Thus, cancer evolution generally requires multiple cycles of macroevolution and microevolution [57,58]. Once cells with new genomes break the systemic constraints to survive, they need to form a large population. During microevolution, associated cancer gene mutations and epigenetic factors can promote population growth [59]. FHIT might bridge macroevolution and microevolution. The stress induced by the abnormal expression of FHIT promotes genomic instability, leading to an increase in aneuploid chromosomes and ssDNA. Decreased expression of the FHIT also suppresses systemic constraints such as cellular checkpoints and induces associated gene mutations to promote microevolution.

### 3.1. Decreased Expression of the FHIT Induces Replication Fork Stagnation

Normal cells have robust mechanisms to maintain correct DNA sequences and genomic stability; however, these mechanisms are often compromised in cancers, leading to damage and variations in cell DNA [60]. Oncogene-induced replication pressure may not be the most common initiating event in the evolutionary development of cancer. Genetic instability and heterogeneity appear in the genome before oncogene activation. This genetic instability usually manifests as mutations in so-called “caretaker” genes when genomic stability is shaken, including variations in chromosome copy number, cell cycle arrest, and point mutations.

The FHIT knockdown leads to an increased proportion of cells with phosphorylated ATR (Ser428) nuclear foci [47], suggesting that the FHIT expression contributes to minimizing and preventing DNA replication. Under normal conditions, the FHIT achieves positive regulation of thymidine kinase 1 (TK1) by promoting ribosome binding to the translation region of TK1 mRNA, thus enhancing the translation of downstream regions. The FHIT limits TK1 protein degradation to mitosis and then supports DNA synthesis of deoxythymidine triphosphate (dTTP) through a clearance pathway of TK1 activity [47]. In proliferating cells, TK1 maintains a stable dTTP pool in proliferating cells with ribonucleotide reductase and thymidylate synthase (TS) [61]. TK1 is regulated in a cell cycle-dependent manner, which is generally expressed in low amounts in the G1 phase. In contrast, the expression of TK1 is significantly increased as cells are prepared to enter the S phase, which help in biosynthesis with the dTTP pool [62]. High expression levels of TK1 protein are required throughout the S phase and G2 phase to ensure the sufficient production of dTTP [63]. When mitosis ends, TK1 is rapidly degraded to prevent the overproduction of dTTP. The FHIT deficiency leads to a decrease in TK1 expression and causes a continuous decrease in dTTP [64]. This leads to site-specific chromosomal instability and polymerase arrest, which affects DNA replication and impedes replication fork progression, promoting genomic instability and further oncogenic transformation.

### 3.2. Replication Stress Causes Chromosome Instability

Under replication stress caused by a decrease in the FHIT expression, levels of spontaneous DNA damage increased in almost all cell types [65,66]. The FHIT knockdown in cells with normal FHIT expression increases spontaneous sister chromatid exchange (SCE) [67]. Such an increase in chromosomal instability can be induced only by the loss of FHIT protein activity in epithelial cells, even without exogenous genotoxic stress. *FHIT* deletion in rat kidney epithelial cells results in a more than two-fold higher rate of CFS breaks, along with an increased frequency of asymmetric sister replication forks outward from a common origin [68]. Mutations and transformation frequencies are also approximately two- to five-fold higher in *FHIT*-deficient cells than in cells with normal FHIT expression. FHIT^-/-^ mouse kidney cells and mouse embryonic fibroblasts (MEF) also show increased insertions/deletions and aneuploid chromosomes, including chromosomal gain or loss [61]. The average tail moment of FHIT-deficient cells is significantly increased compared to siRNA-control cells, as measured via a single-cell gel electrophoresis assay or neutral comet assay, suggesting that reduced FHIT expression leads to spontaneous DSBs [47]. These DSBs preferentially target other CFSs, resulting in allelic imbalance [35]. The genome appears more fragilely broken and more altered [67]. As time passes, many genomic alterations accumulate, generating significant mutational diversity and cellular heterogeneity. Spontaneous DSBs are highly severe forms of DNA damage [69]. Although they can be repaired via NHEJ or homologous recombination, incorrect repairs might occur via introducing clusters of point mutations, small insertions, and deletions, or leading to non-random clusters of mutations [60,70,71].

### 3.3. Decreased Expression of the FHIT Stops Cellular Checkpoints

Cells need to block cell cycle progression and coordinate replication forks by activating replication checkpoints in S-phase to reduce DSBs induced by replication stress. ATMRad3-related gene (ATR) and checkpoint kinase 1 (Chk1) are key sites for S-phase replication checkpoints, and ATR localizes stalled forks and phosphorylates multiple targets, such as Chk1. Phosphorylated Chk1 is activated to phosphorylate its targets through the Chk1 and cell division cycle protein 25 (CDC25) pathway. It adjusts cyclin dependent kinase 2 (CDK2)-cell cycle protein E kinase to execute S-phase checkpoints [72]. However, the enhanced expression of phosphorylated-Chk1 after the FHIT knockdown demonstrates that the S phase checkpoint is not activated, leading to the continued accumulation of defective cells [47,73]. Aberrant expression of the FHIT induces a level of genomic replication stress that leads to the generation of many DSBs and intrinsic errors of the DNA replication machinery, which increases the mutation rate in the cancer genome and provides opportunities for mutations to accumulate.

## 4. Decreased Expression of the FHIT Catalyzes APOBEC3B Hypermutation

### 4.1. APOBEC3B Requires Co-Factors to Promote Hypermutation

APOBEC3B is an endogenous mutagen that induces mutations by specifically targeting cytosine to uracil ssDNA deamination, most commonly C>T and C>G mutations [74,75]. Kataegis and kyklonas are two clustered somatic mutational events highly associated with the APOBEC3 family [76]. Kataegis is a more-extended strand coordination event arising from multiple mutations [77,78], while kyklonas means a co-occurred event of kataegis and extrachromosomal DNA (ecDNA) in the APOBEC3-induced samples. Uracil glycosylase (UNG) is a critical molecule in the nucleotide excision repair pathway [5,10]. UNG recognizes and excises APOBEC3B-induced C>U mutations [79]. Then it forms purine/pyrimidine resolving sites and triggers nucleic acid chain hydrolysis to eliminate mismatches [80,81], thereby maintaining genomic stability. We demonstrate that IL-6 *trans*-activates the expression of APOBEC3B and *trans*-inactivates the expression of UNG, thus unbalancing APOBEC3B/UNG and facilitating cancer evolution and development [80]. UNG is classified into intranuclear (UNG2) and mitochondrial (UNG1). UNG2 is more active and has a more substantial effect on ssDNA [82]. Although there are many C>T and C>G mutations in the genome, increased expression of APOBEC3B is not associated with a high mutational load in cancer [83]. This finding suggests that APOBEC3B may require the other factors together to achieve hypermutation.

### 4.2. Mutation Characteristics Caused by Decreased Expression of FHIT Are Similar to APOBEC Signature

There are multiple mutations in the human cancer genome. Abnormal FHIT expression is a molecular determinant of the “Catalog of Somatic Mutations in Cancer mutational signature 5” [68], which mainly occurs in the early stages of various cancers and accelerates carcinogenesis through oncogenic exposure. Tumors with low FHIT expression have a higher mutational load than tumors with typical FHIT expression. A nearly four-fold increase in total single base substitutions is evident in the FHIT-deficient liver tissues [84]. In addition, there is a significant increase in C>T mutations and slight elevation in the C>A, C>G, and T>A mutations [85]. The expression of APOBEC3B in glioma cells is significantly opposite to that of the FHIT. Data from the adenocarcinoma of the lung in The Cancer Genome Atlas (TCGA) show frequent reduction of the FHIT expression in combination with a high signature of APOBEC3B. Synergic action between the FHIT and APOBEC3B in cancer has been observed in several cancers. The low or complete lack of FHIT expression appears to be a prerequisite for APOBEC3B hypermutation [86]. Several mutations with similar characteristics to APOBEC3B occurred in FHIT-deficient tumors, suggesting that FHIT inactivation acts synergistically with APOBEC3B to promote APOBEC3B-related hypermutation.

### 4.3. FHIT Provides Optimal Substrate for APOBEC3B Hypermutation

Typically, ssDNA is an optimal substrate for the AID/APOBEC3 cytidine deaminases, which is 200–300 times more efficient than dsDNA [5,74]. Slowed or stalled replication forks due to aberrant FHIT expression may be a key to triggering prolonged exposure of ssDNA to the cellular environment. The FHIT deficiency sensitizes cells to DNA damage, promotes genomic instability, and creates a sustained climate of DNA damage to further oncogenic transformation. In early lesions of many types of cancer, the FHIT protein inactivation induces replication stress. Uncoupling of polymerase and helicase activities due to an obstruction in the replication stress produces breaks in dsDNA, leading to increased ssDNA substrate. The accumulation of ssDNA provides a suitable substrate for APOBEC3B enzyme activity [87]. APOBEC3B then catalyzes APOBEC3B-mediated hypermutation formation and clonal amplification, leading to cancer development and progression. Although most mutations in cancer are “passengers” and do not critically affect the process of cancer evolution and development, the positive selected mutations frequently caused by abnormal FHIT expression tend to be a “driver”. The abnormal FHIT expression facilitates the APOBEC3B-driven hypermutations in cell cycle- and metabolism-related genes, leading to the emergence of APOBEC3-associated mutational features and cellular carcinogenesis.

**Figure 2:**
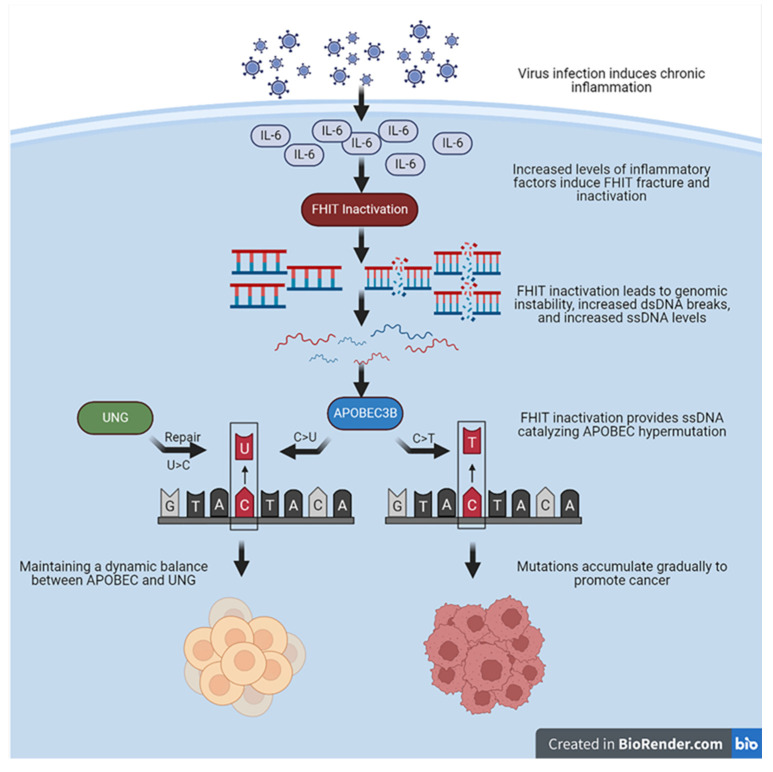
Mechanistic assumptions between FHIT, UNG, and APOBEC3B.

A double-stranded structural state mainly protects the human genome. With the UNG repair response, the chance of mutations catalyzed by APOBEC3’s attack on single strands formed by transcriptional activity in the short term is low or at a low-frequency level. However, when the inflammatory response is chronic, either the mutations directly caused by APOBEC3B or the errors accumulated by the long-term high-frequency repair of UNG will become the basis for the generation of somatic mutations required for cancer evolution. Nevertheless, the mechanistic relationship between APOBEC3B, the FHIT, and UNG remains to be clarified. Further studies are still needed to explore the function of the FHIT in the interconnection between APOBEC3B and UNG. The FHIT and UNG appear to be suppressed from two distinct perspectives for APOBEC3B mutations. UNG recognizes and excises APOBEC3s-induced C>U mutations while regular expression of the FHIT decreases the cytidine deaminase activity of APOBEC3B by maintaining genomic stability and reducing ssDNA. Based on the above evidence, we hypothesize that during chronic infection-related chronic inflammation or chronic inflammation produced by other causes, IL-6 and other proinflammatory cytokines might inactivate or downregulate the expression or function of the FHIT. An inactivated FHIT leads to genomic instability and increases the chance of dsDNA breaks to ssDNA. The proinflammatory cytokines also *trans*-activate the expression of APOBEC3B and *trans*-inactivate the expression of UNG. Upregulated APOBEC3B prefers to edit ssDNA and accumulate the APOBEC3 mutation signature in human genome and ecDNA, which promote cancer evolution and development in the proinflammatory TME (Figure 2). Hypoxia and demethylation in the TME may facilitate the retro-differentiation of the mutated cells into cancer initiation cells. Further studies are needed to address the mutation features in the FHIT-deficient pre-tumor lesion and tumor models with distinct levels of APOBEC3B and UNG.

## 5. Abnormal Expression of FHIT Induces EMT and Inhibits Apoptosis

### 5.1. FHIT Deficiency Affects the Mitochondria-Mediated Apoptosis Pathway

When exogenous and endogenous factors induce DNA damage, the damage checkpoints respond in two pathways [73]. First, a barrier is formed to mediate checkpoint arrest in tumor cells and temporarily or permanently arrest cells in the cell cycle. Second, the apoptotic pathway is activated to clean up cells that proliferated abnormally or suffered DNA damage. Potentially oncogenic mutations cannot be perpetuated, thus preventing tumor cells from continuing to develop and causing cancer. Two main pathways promote apoptosis. One is the mitochondria-mediated endogenous apoptotic pathway, an intrinsic pathway under the control of the Bcl-2 family [88,89]. The release of cytochrome C and Smac/Diablo is induced from the mitochondrial intermembrane space. Cytochrome C can form an apoptosome with Apaf-1, the caspase-9 precursor, and ATP/dATP, which convenes and activates caspase-3. Caspase-3 then triggers a cascade of caspases, leading to apoptosis. Second, during oxidative stress, the FHIT interacts with ferric chelate reductase in mitochondria to increase reactive oxygen species (ROS) and induce caspase-3 activation and apoptosis [65]. Furthermore, the FHIT increases mitochondrial calcium release to promote apoptosis [90]. The FHIT protein also induces apoptosis in cancer cells by altering the mitochondrial transmembrane potential and enhancing cytochrome C efflux from mitochondrial cells [49]. The execution of this apoptotic program is not blocked by Bcl-2 or Bcl-x(L) over-expression [91]. However, the FHIT-deficient cancer cells can escape ROS overproduction and ROS-induced apoptosis, leading to the continued proliferation of cancer cells and eventually carcinogenesis. When the particular DNA used to control normal cell differentiation is mutated and is not repaired in time, the cells may become malignant proliferating cells. The cells are no longer under the organism’s control and divide in an uncontrolled manner. In MEF, FHIT^-/-^ cells undergo chromosomal alterations, leading to the amplification of the MDM2 and rapid immortalization of the MEF [25]. This is exceptionally important for cancer evolution, as genomic instability in the FHIT-deficient cells leads to rapid proliferation and immortalization, allowing cancer cells to proliferate and grow indefinitely, thus allowing mutations and damage to be passed on over time, and promoting cancer evolution.

### 5.2. Decreased Expression of the FHIT Promotes Reverse Differentiation of Cancer Cells

Cancer evolution is often accompanied by reverse differentiation, and reverse development from differentiated cells to undifferentiated cells [5,10]. The EMT is a hallmark event of the reverse developmental process. EMT, with stem cell property, confers the ability to metastasize and invade suitable tissues. EMT also reduces apoptosis and cellular senescence, and promotes immunosuppression, catalyzing cancer development and progression. Regular expression of the FHIT helps inhibit the development and progression of human lung cancer by activating the miR-30c-targeted metastatic genes Metadherin, high-migration group AT-hook 2, vimentin, and fibronectin, helping inhibit EMT and cancer cell metastasis [92]. The FHIT silencing in bronchial epithelial cells increases Slug-dependent cell invasion by regulating EGFR-induced overexpression of two primary EMT-related targets, MMP-9 and Vimentin [93]. At the same time, activation of inflammatory factors releases pro-inflammatory factors that lead to the phosphorylation of STAT3 [94]. Phosphorylated STAT3 at the Tyr705 site also enter the nucleus to bind to specific fragments on the EMT transcription factor promoter and activate the EMT pathway [95]. Activated STAT3 *trans*-activates DNA methyltransferases DNMT1 and DNMT3B [96] and recruits them to the promoter region of the FHIT, leading to increased levels of the FHIT promoter CpG methylation and reduced FHIT expression.

**Figure 3:**
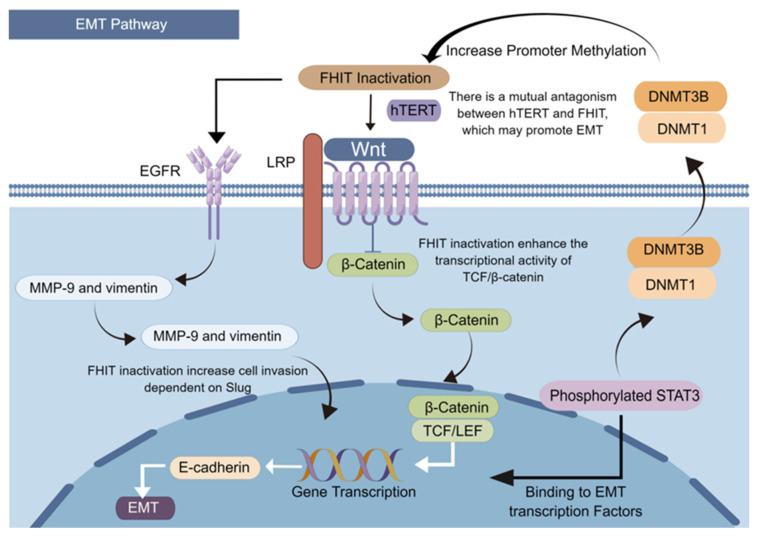
FHIT loss induces epithelial–mesenchymal transition.

The Wnt-β-catenin signaling pathway mediated by β-catenin is a transduction pathway that induces critical signals for EMT production in epithelial tissues; the dysregulation of this pathway is associated with various cancers. The binding of the FHIT to the C-terminal domain of β-catenin inhibits the transcription of target genes such as cell cycle protein D1. The loss or reduction of the FHIT expression in cells will enhance the transcriptional activity of TCF/β-catenin, promoting the invasive ability [93]. Telomerase reverse transcriptase (hTERT)-induced telomerase activation confers unlimited proliferative potential to cancer cells by stabilizing their telomere length [97]. It also interacts with β-catenin and strongly amplifies their transcriptional output, thereby stimulating EMT and cancer cell stemness [98]. Normal human cells do not express hTERT; however, hTERT levels are elevated and negatively correlated with FHIT expression in cancers. This finding suggests a mutual antagonism between hTERT and the FHIT, and that hTERT is likely an essential component of the FHIT deficiency promoting EMT (Figure 3).

## 6. Conclusions

The developmental process of cancers includes genetic damage and epigenetic changes triggered by exposure to carcinogenic factors, gene mutation and selection, infiltration of inflammatory cells such as neutrophils in the TME, cancer development, and even metastasis. At the stage of pre-cancerous lesion, the FHIT gene damage caused by exogenous carcinogenic exposure, endogenous replication pressure, and methylation at the promoter region leads to replication pressure and genomic instability, facilitating the generation of macroevolution and dsDNA breaks to ssDNA. The generation of ssDNA by the FHIT absence also provides ample opportunities for APOBEC3B to generate mutations within the genome, which induces APOBEC3B-related hypermutation, a process termed microevolution, characterized as “mutation-selection-adaptation”. Thus, the FHIT damage might bridge macroevolution and microevolution, especially at the early stage of carcinogenesis. The mutated cells take part in the construction of proinflammatory TME and then adapt to it. With the accumulation of positively selected driver mutations in the TME, the mutated cells are retro-differentiated and eventually acquire “stemness” through the EMT-like pathway, causing evolutionary heterogeneity to gain a survival advantage and promoting cancer development from pre-cancerous lesions. The FHIT damage also facilitates the retro-differentiation of the mutated cells. Thus, the role of the FHIT is indispensable for optimizing the theory of Cancer Evo-Dev. Currently, studies on the FHIT remain very limited. Understanding how external factors affect the FHIT in early inflammatory or viral infection settings and how the FHIT inactivation facilitates cancer evolution and development are important for developing suitable prophylactic and therapeutic options for different cancer types. This pursuit might lead to developing clinical targets to maintain genomic integrity via stabilizing the FHIT, thus preventing transformation from precancerous lesions into cancer.
